# The Role of NOX2-Derived Reactive Oxygen Species in the Induction of Endothelin-Converting Enzyme-1 by Angiotensin II

**DOI:** 10.3390/antiox13040500

**Published:** 2024-04-22

**Authors:** Michael Adu-Gyamfi, Claudia Goettsch, Julian Kamhieh-Milz, Lei Chen, Anna Maria Pfefferkorn, Anja Hofmann, Coy Brunssen, Gregor Müller, Thomas Walther, Muhammad Imtiaz Ashraf, Henning Morawietz, Janusz Witowski, Rusan Catar

**Affiliations:** 1 Department of Nephrology and Medical Intensive Care, Charité-Universitätsmedizin Berlin, 13353 Berlin, Germany; michael.adu-gyamfi@charite.de (M.A.-G.); lei.chen1991@gmail.com (L.C.); 2 Division of Vascular Endothelium and Microcirculation, Department of Medicine III, University Hospital, Faculty of Medicine Carl Gustav Carus, Technische Universität Dresden, 01307 Dresden, Germany; cgoettsch@ukaachen.de (C.G.); anja.hofmann2@uniklinikum-dresden.de (A.H.); coy.brunssen@ukdd.de (C.B.); gregor.mueller@uniklinikum-dresden.de (G.M.); henning.morawietz@uniklinikum-dresden.de (H.M.); 3 Department of Internal Medicine I-Cardiology, Medical Faculty, RWTH Aachen University, 52072 Aachen, Germany; 4 Institute of Transfusion Medicine, Charité-Universitätsmedizin Berlin, 13353 Berlin, Germany; julian.milz@charite.de; 5 Department of Nephrology, The Fifth Affiliated Hospital, Sun Yat-sen University, Zhuhai 519082, China; 6 Department of Surgery, Experimental Surgery, Charité-Universitätsmedizin Berlin, 13353 Berlin, Germany; anna.pfefferkorn@gmx.de (A.M.P.); muhammad-imtiaz.ashraf@charite.de (M.I.A.); 7 Division of Vascular and Endovascular Surgery, Department of Visceral, Thoracic and Vascular Surgery, University Hospital, Faculty of Medicine Carl Gustav Carus, Technische Universität Dresden, 01307 Dresden, Germany; 8 Medical School Berlin (MSB), 14197 Berlin, Germany; t.walther@ucc.ie; 9 Xitra Therapeutics GmbH, 17489 Greifswald, Germany; 10 Department of Pathophysiology, Poznan University of Medical Sciences, 60-535 Poznan, Poland; 11 Berlin Institute of Health, 10178 Berlin, Germany

**Keywords:** angiotensin II, ECE-1, endothelial cells, endothelin-1, NADPH oxidase, Oct-1, reactive oxygen species

## Abstract

Endothelin-1 is a key regulator of vascular tone and blood pressure in health and disease. We have recently found that ET-1 production in human microvascular endothelial cells (HMECs) can be promoted by angiotensin II (Ang II) through a novel mechanism involving octamer-binding transcription factor-1 (Oct-1), NADPH oxidase-2 (NOX2), and superoxide anions. As the formation of bioactive ET-1 also depends on endothelin-converting enzyme-1 (ECE-1), we investigated the transcriptional regulation of the *ECE1* gene. We found that exposure of HMECs to Ang II resulted in a concentration- and time-dependent increase in *ECE1* mRNA expression. Pharmacological inhibition of ECE-1 reduced Ang II-stimulated ET-1 release to baseline values. The effect of Ang II on *ECE1* mRNA expression was associated with Oct-1 binding to the *ECE1* promoter, resulting in its increased activity. Consequently, the Ang II-stimulated increase in *ECE1* mRNA expression could be prevented by siRNA-mediated Oct-1 inhibition. It could also be abolished by silencing the *NOX2* gene and neutralizing superoxide anions with superoxide dismutase. In mice fed a high-fat diet, cardiac expression of *Ece1* mRNA increased in wild-type mice but not in *Nox2*-deficient animals. It can be concluded that Ang II engages Oct-1, NOX2, and superoxide anions to stimulate *ECE1* expression in the endothelium.

## 1. Introduction

ET-1 (endothelin 1) is a parent compound of a family of endothelial cell-derived peptides with potent and long-lasting vasoconstrictor activity. Extensive studies have clearly demonstrated the key role of endogenous ET-1 in the maintenance of vascular tone, blood pressure, and tissue perfusion. However, if dysregulated, ET-1 may also exert pathologic effects, as observed in systemic and pulmonary hypertension, heart failure, and chronic kidney disease. These effects are largely related to excessive levels of ET-1, which promote endothelial dysfunction and the progression of atherosclerosis. As a result, pharmacological modification of ET-1 activity has been proposed as a therapeutic option. Large efforts in this respect have, however, produced mixed results so far [1]. This is because of the complexity of endothelin system regulation and its effects, which are dependent on the pathophysiological context.

ET-1 is produced by endothelial cells lining all types of blood vessels. Its production and release are multi-step processes that occur both constitutively and in response to many stimuli. The synthesis of prepro-ET1, a precursor protein, is regulated predominantly at a transcriptional level and may involve many transcription factors [2]. The final step in prepro-ET1 conversion into mature and active ET-1 is mediated by endothelin-converting enzyme-1 (ECE-1), a protease bound to the membranes of secretory vesicles and Weibel-Palade granules from which mature ET-1 is released [1]. The regulation of ECE-1 itself is not fully understood. In humans, differential splicing of mRNA transcribed from a single gene under the control of alternative promoters can generate four ECE-1 isoforms (ECE-1a–d) [3,4]. They have similar enzymatic properties but differ in subcellular localization and tissue distribution [5,6]. Of these, ECE-1c appears to be predominant and the most abundant. Global deletion of *Ece1* in mice results in severe craniofacial and cardiovascular abnormalities and embryonic lethality [7]. It is not clear, however, whether these effects result only from the absence of bioactive ET-1, since ECE-1 may also cleave substrates other than endothelins [8]. Interestingly, in the absence of ECE-1, some levels of mature ET-1 can probably be generated by the proteolytic activity of chymase [9,10].

We have recently characterized a pathway by which angiotensin II (Ang II) can stimulate endothelial ET-1 production. It involves the induction of nicotinamide adenine dinucleotide phosphate oxidase 2 (NOX2) expression and the production of superoxide anions that activate the ET1 promoter and increase *ET1* mRNA expression and protein release [11]. In the present study, we investigated the effect of Ang II on ECE-1.

## 2. Methods

### 2.1. Materials

Unless stated otherwise, all reagents were from Merck (Darmstadt, Germany). Inhibitors used were: CGS35066, an ECE-1 inhibitor (Tocris Bioscience/Bio-Techne; Wiesbaden, Germany); valsartan, an angiotensin receptor type I (AT1) antagonist (Sigma-Aldrich; Schnelldorf, Germany, Merck, Darmstadt, Germany); PD-184352, an inhibitor of extracellular signal-regulated kinases (ERK) (Cell Signaling Technology, Danvers, MA, USA); and PEG-SOD, a superoxide dismutase-polyethylene glycol conjugate (Sigma-Aldrich^®^, Merck). Their effective concentrations were determined in preliminary experiments.

### 2.2. Cell Culture

Human microvascular endothelial cells (HMECs) of the dermal HMEC-1 line (catalogue no. CRL-3243) were purchased from ATCC^®^ (Manassas, VA, USA) and grown in MCDB131 medium (ThermoFisher Scientific, Waltham, MA, USA) supplemented with 10% (*v*/*v*) Gibco^TM^ fetal calf serum (FCS), 2.5 mmol/L glutamine, 100 U/mL penicillin, 100 µg/mL streptomycin, 2 pmol/L hydrocortisone, and 5 ng/mL epidermal growth factor (EGF).

### 2.3. Immunoassays

Human ET-1 was measured using the immunoassay kit from R&D Systems (Bio-Techne; Wiesbaden, Germany) following the manufacturer’s instructions.

### 2.4. Gene Expression Analysis

Expression of target genes was assessed with reverse transcription and quantitative PCR (RT-qPCR). RNA extraction, reverse transcriptions, and PCR were performed exactly as described previously [11]. Transcript levels were normalized to endogenous controls (human β2-microglobulin or mouse *Gapdh*) and presented in arbitrary units (AU). The PCR primers used are listed in Table 1.

### 2.5. Promoter Analysis, Plasmid DNA Constructs

The potential transcription factor binding sites in the human *ECE1* promoter sequence from −609 to −1107 (GenBank NC_000001.11) were searched using Alibaba2—Gene Regulation (http://gene-regulation.com/pub/programs/alibaba2/ accessed on 23 June 2023). Genomic DNA was isolated using an Isol-RNA lysis solution (5′-Prime, Hamburg, Germany). It was then used to construct luciferase reporter plasmids for *ECE1* promoter fragments (ECE1_2000, _1108, _608, and _108) using PCR with appropriate primers. The pGL4.10 luciferase reporter plasmid backbone (Promega, Madison, WI, USA) was constructed using the Infusion Cloning Kit (Clontech, Takara Bio, San Diego, CA, USA). The length of the promoter fragments was verified by restriction digest and sequencing (LGC Genomics, Berlin, Germany).

### 2.6. Cell Transfections

Cells at 70–80% confluence were starved overnight in medium with a reduced concentration of FCS (0.5%) and transfected with *ECE1* promoter plasmids and reference pRL-TK Renilla plasmids using the TurboFect transfection reagent (Takara, San Jose, CA, USA) according to the manufacturer’s instructions. Luciferase activity of *ECE1* promoter plasmids was measured using a luminometer (Fluostar Optima, BMG Labtech, Ortenberg, Germany) and normalized to the background activity of Renilla luciferase from co-transfected control vectors.

Transient transfections of HMECs with siRNAs targeting *NOX1* (sc-43939), *NOX2* (sc-35503), *NOX4* (sc-41586), *OCT1* (sc-42552), or a scrambled siRNA control (sc-37007) (all from Santa Cruz Biotechnology, Heidelberg, Germany) were performed as per the manufacturer’s instructions. The efficacy of siRNAs in down-regulating the expression of corresponding mRNAs and proteins in Ang II-treated HMECs was analyzed by RT-qPCR and Western blotting as described in detail elsewhere [11] and presented in the Supplementary Materials.

### 2.7. Electronic Shift Mobility Assay (EMSA)

Nuclear extracts were obtained using the NE-PER Nuclear and Cytoplasmic Extraction reagent (ThermoFisher Scientific, Darmstadt, Germany). A specific oligonucleotide probe for Oct-1 binding was labeled with Biotin 3’ End DNA labeling reagent (ThermoFisher Scientific, Darmstadt, Germany). The probe sequence was as follows (with the corresponding region of the ECE1 promoter given in brackets): *OCT-1*, 5′-CAAATCCCAAATATAGTCAGGACT-3′ (-648 to -671). EMSA was performed as described earlier in detail [11], and the results were visualized following electrophoresis on 6% non-denaturing polyacrylamide gels and detection with a Light Shift chemiluminescent EMSA Kit (ThermoFisher Scientific, Darmstadt, Germany).

### 2.8. Animal Studies

Animal tissue analysis was performed on archival samples collected during a previous study, as described in detail [11]. Briefly, samples of the hearts were obtained from wild-type (WT) C57BL/6J mice or *Nox2*^−/−^ animals after 10 weeks of feeding ad libitum with either a standard laboratory diet or a high-fat diet (both from Ssniff Spezialdiäten GmbH, Soest, Germany). All procedures involving animals were performed in accordance with the “Guide for the Care and Use of Laboratory Animals” (Institute for Laboratory Animal Research of the National Research Council, USA) and approved by the local institutional committee for ethics in animal research (decisions no. 24D-9168.24-1-2005-14 and 24-9168.24-1-2003-12).

### 2.9. Statistical Analysis

Statistical analysis was performed using GraphPad Prism version 10.1.2 (GraphPad Software, San Diego, CA, USA). The data were analyzed for normality using the Shapiro–Wilk test. Normally distributed data were analyzed without assuming equal variances using either a t-test with Welch’s correction or a Brown–Forsythe and Welch analysis of variance (ANOVA) followed by a Dunnett T3 test for multiple comparisons. When required, repeated measures ANOVA or two-way ANOVA (both with Šidák’s correction) were used. Abnormally distributed data were analyzed with the Kruskal–Wallis test. Data were expressed as means ± SD. A *p*-value < 0.05 was considered statistically significant.

## 3. Results

### 3.1. Role of ECE-1 in ET-1 Production by HMECs Stimulated with Ang II

**Figure 1:**
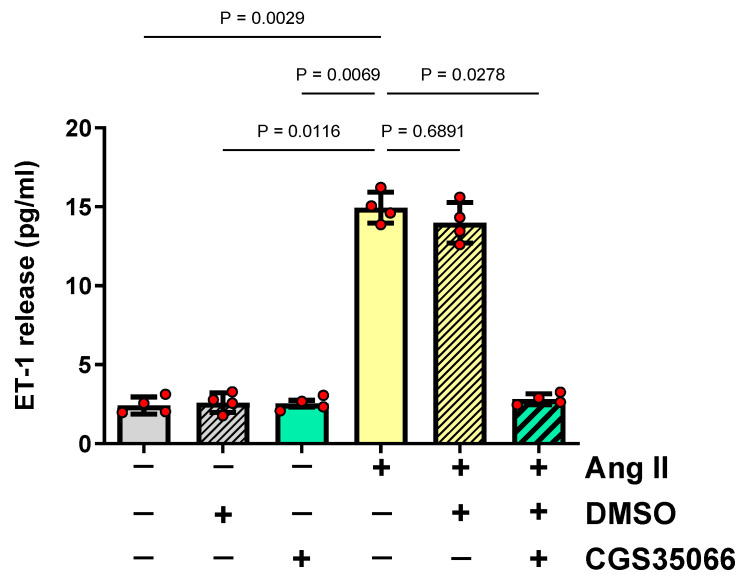
**The role of ECE-1 in ET-1 production by Ang II-stimulated HMECs.** HMECs were pre-treated for 1 h with either a specific ECE-1 inhibitor, CGS35066 (1 nmol/L), or an equal volume of vehicle (DMSO), stimulated with Ang II (1 μmol/L) for 24 h, and then analyzed for the released amount of ET-1 (n = 4). All data were analyzed with an ANOVA.

To determine whether ECE-1 is necessary for Ang II-induced ET-1 production, HMECs were first pre-incubated with a specific ECE-1 inhibitor, CGS35066, and then stimulated with Ang II at a concentration known to stimulate ET-1 secretion [11]. This experiment showed that the blockade of ECE-1 completely abolished ET-1 release by HMECs (Figure 1).

### 3.2. Induction of ECE1 Expression by Ang II in HMECs

**Figure 2:**
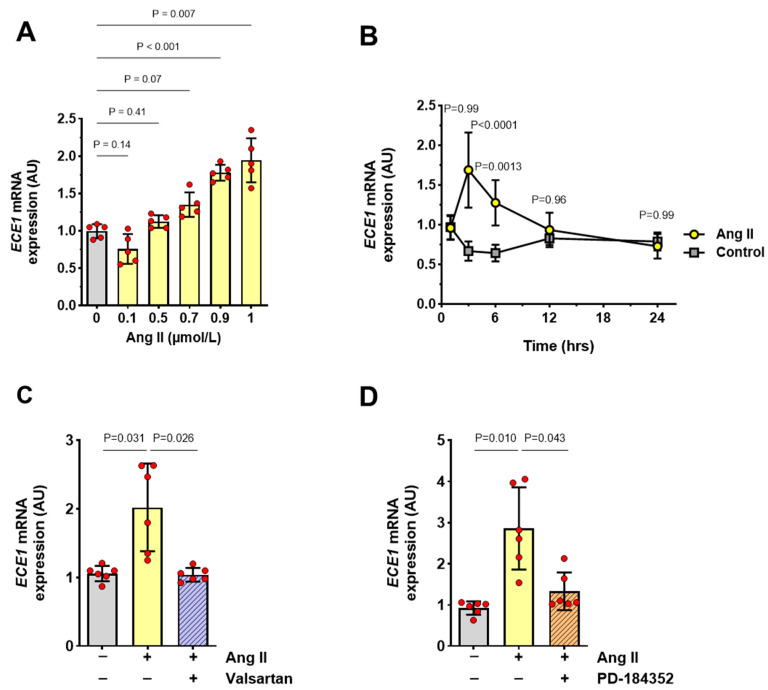
***ECE1* expression by HMECs stimulated with Ang II.** (**A**) Dose effect of Ang II on *ECE1* mRNA expression in HMECs. Cells were incubated with increasing concentrations of Ang II for 3 h (n = 5). (**B**) Time effect of Ang II (1 µmol/L) on *ECE1* mRNA expression (n = 4). (**C**,**D**) HMECs were pre-treated for 1 h with (**C**) an Ang II receptor blocker, valsartan (10 μmol/L), or (**D**) an ERK inhibitor, PD-184352 (1 μmol/L), and then stimulated with Ang II (1 µmol/L) for 3 h and analyzed for *ECE1* mRNA expression (n = 6). All data were analyzed with ANOVA and Šidák’s multiple comparisons test.

Exposure of HMECs to Ang II resulted in a concentration- and time-dependent increase in *ECE1* mRNA expression. The greatest effect was caused by Ang II at a concentration of 1 µmol/L (Figure 2A). The resulting increase in *ECE1* mRNA expression peaked at 3 h and then returned to baseline values (Figure 2B).

Then, the effect of Ang II on *ECE1* mRNA expression was studied in cells pre-treated either with valsartan, an antagonist of angiotensin receptor type I, or with PD-184352, an inhibitor of extracellular signal-regulated kinases, as these agents were previously found to block the effect of Ang II on *ET1* mRNA expression [11]. Exposure of HMECs to valsartan (Figure 2C) or PD-184352 (Figure 2D) reduced *ECE1* mRNA expression to control levels.

### 3.3. Effect of Ang II on the ECE1 Promoter in HMECs

**Figure 3:**
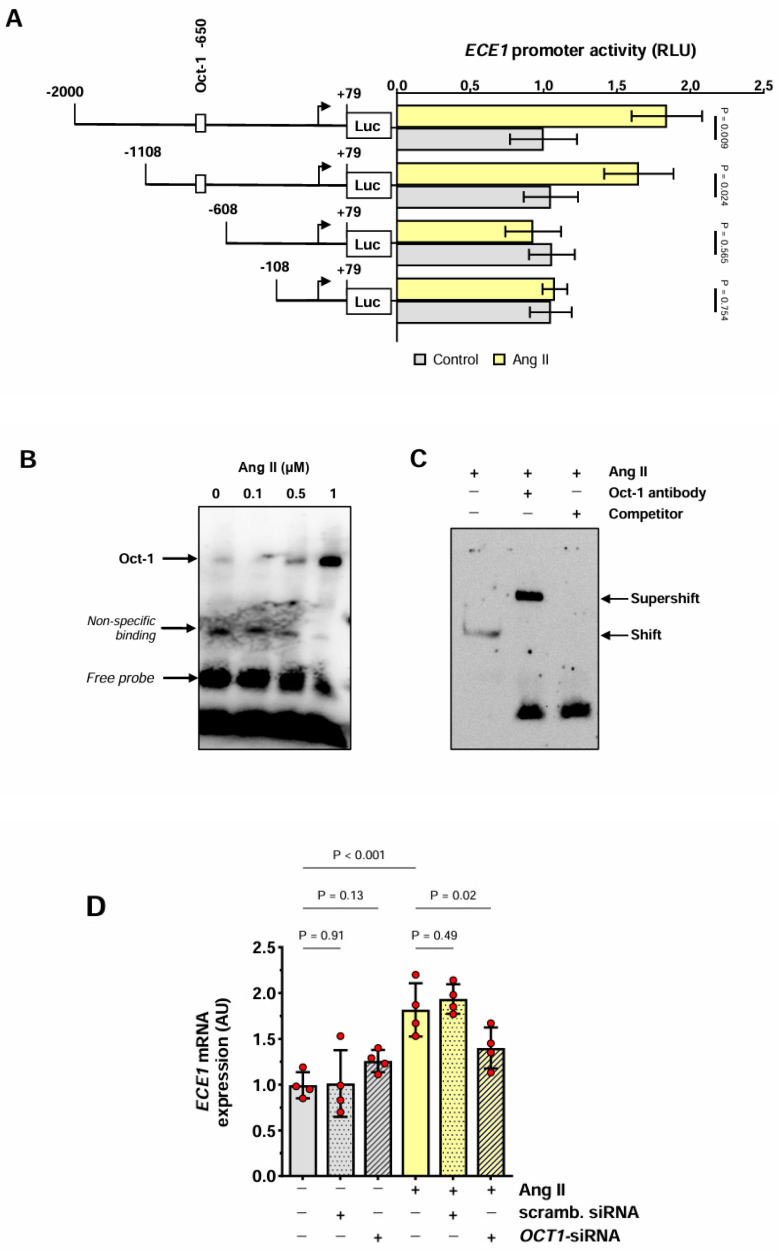
**Effect of Ang II on the *ECE1* promoter.** (**A**) HMECs were transiently transfected with *ECE1* promoter constructs with 5′-deletions of various lengths. After 3 h of stimulation with Ang II (1 μmol/L), cells were assessed for the activity of *ECE1* promoter fragments (n = 4). The data were analyzed with multiple unpaired *t*-tests vs. control cells transfected with the same construct. (**B**,**C**) Nuclear fractions from cells stimulated with Ang II (1 μmol/L) were analyzed by EMSA using a biotin-labeled probe containing the predicted *ECE1* promoter sequence that bound Oct-1. In (**B**), cells were stimulated with increasing concentrations of Ang II as indicated. In (**C**), cells were stimulated with 1 μmol/L of Ang II and EMSA was performed in the presence of either a 100-fold molar excess of unlabeled *ECE1* DNA or an Oct-1-specific antibody. In (**B**,**C**), representative experiments are shown; (**D**) HMECs were transiently transfected with either *OCT1*-specific siRNA or scrambled siRNA (at 10 nmol/L). Subsequently, HMECs were stimulated with Ang II (1 µmol/L) for 3 h and analyzed for *ECE1* mRNA expression (n = 4). The data were analyzed with an ANOVA.

Exposure of HMECs transfected with *ECE1* luciferase reporter gene constructs to Ang II resulted in a significant increase in the full-length *ECE1* promoter activity (Figure 3A). To identify the *ECE1* promoter region responsive to Ang II, its partial deletions were performed. Removal of the fragment containing positions −1108 to −609 eliminated the ability of the *ECE1* promoter to respond to Ang II. The deleted fragment was analyzed in silico and found to contain a potential high-affinity binding site for octamer-binding transcription factor 1 (Oct-1). An electrophoretic mobility shift assay (EMSA) using a biotin-labeled oligonucleotide probe for Oct-1 that corresponded to positions −838 to −862 of the *ECE1* promoter showed the formation of a complex with the nuclear fraction extracted from HMECs treated with Ang II (Figure 3B). To demonstrate the specificity of Oct-1 binding, EMSA was performed either in the presence of an anti-Oct-1 antibody or after the addition of an unlabeled oligonucleotide probe in a 100-fold excess. It was found that under these conditions, the DNA-protein complex was “shifted” or competed away, respectively (Figure 3C).

To confirm the involvement of Oct-1 in the regulation of *ECE1* gene expression, HMECs were stimulated with Ang II following the transfection with either *OCT1*-silencing siRNA or scrambled control siRNA. This intervention led to a reduction in *ECE1* mRNA expression in cells treated with *OCT*-1 siRNA but not control siRNA (Figure 3D).

### 3.4. Role of NOX2-Derived Superoxide Anions in the Induction of ECE1 Expression

**Figure 4:**
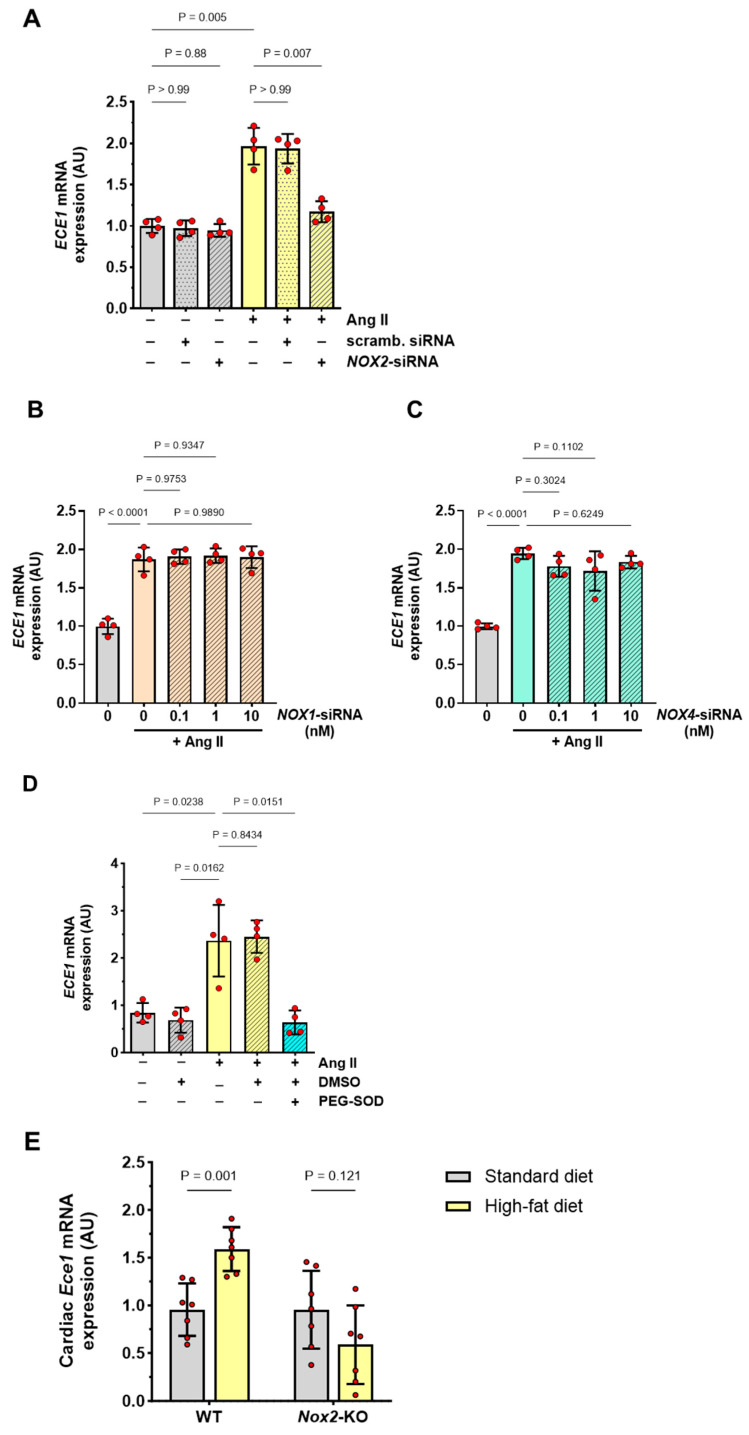
**Role of NOX2 for ECE1 expression.** (**A**–**C**) HMECs were transiently transfected with *NOX2*-specific siRNA or scrambled siRNA (both at 10 nmol/L) (**A**), *NOX1*-siRNA (0.1–10 10 nmol/L) (**B**), or *NOX4*-siRNA (0.1–10 10 nmol/L) (**C**), and then stimulated with Ang II (1 µmol/L) for 3 h and analyzed for *ECE1* mRNA expression (n = 4); (**D**) HMECs were treated for 3 h with Ang II (1 µmol/L) in the presence of 10 U/mL PEG-SOD or DMSO vehicle alone, and analyzed for *ECE1* mRNA expression (n = 4). The data in A and B were analyzed with ANOVA (n = 4). (**E**) Cardiac expression of *Ece1* mRNA in WT mice and *Nox2*-KO mice fed for 10 weeks with either a regular laboratory diet or a high-fat diet. The data were analyzed using a two-way ANOVA.

Since Ang II-induced Oct-1 was found to regulate ET-1 production in HMECs by controlling *NOX2* expression and NOX2-dependent superoxide anion production [11], we examined whether a similar mechanism existed in the regulation of *ECE1* expression. We first analyzed the effect of *NOX2* gene silencing on *NOX2* mRNA and NOX2 protein expression in Ang II-treated HMECs. Consistent with what we have observed previously [11], we found them both to be reduced by *NOX2*-siRNA in a dose-dependent manner (Supplementary Figure S1). We next assessed HMECs for *ECE1* expression and found that the use of *NOX2*-siRNA, but not control siRNA, reduced Ang II-induced *ECE1* mRNA expression to basal levels (Figure 4A). To check whether this effect could also be mediated by other NOX isoforms, siRNAs targeting either *NOX1* or *NOX4* were used. These siRNAs were effective in reducing the expression of the respective targets at both mRNA and protein levels (Supplementary Figure S2). In contrast to *NOX2*-siRNA, however, these treatments did not reduce the levels of Ang II-induced *ECE1* mRNA (Figure 4B,C).

We then analyzed the effects of superoxide anion neutralization and found that in the presence of PEG-SOD, Ang II-induced *ECE1* mRNA expression was abolished (Figure 4C). Finally, we compared *Ece1* mRNA expression in the hearts of WT mice and *Nox2*-deficient mice. We found there was no difference in this respect between WT and *Nox2*-defient mice receiving a standard laboratory diet. However, when mice were fed a high-fat diet, the cardiac expression of *Ece1* mRNA increased significantly in WT mice but not in *Nox2*-deficient mice (Figure 4D).

## 4. Discussion

In the present study, we have extended our earlier observations and shown that, in addition to driving ET-1 synthesis, Ang II stimulates the expression of ECE-1, a key component of the ET-1 secretion pathway. In this respect, Ang II appears to utilize the same signaling pathway as described previously for ET-1 [11]. It involves the Oct-1-mediated induction of *NOX2* and subsequent superoxide anion production. Accordingly, inhibition of either Oct-1 or NOX2 with siRNAs, as well as neutralization of superoxide by SOD, reduces Ang II-stimulated *ECE1* expression. Thus, by engaging the same regulatory axis of Oct-1 and NOX2, Ang II promotes simultaneously the transcription of both the *ET1* gene and the *ECE1* gene, ensuring efficient ET-1 production and activity. This mechanism appears to control a regulatory, rather than constitutive pathway of ET-1 release, as we did not observe any inhibitory effect of Oct-1/NOX2 blockade on basal *ET1* expression by HMECs. It remains to be established whether such a concordant regulation of *ET1* and *ECE1* occurs also in response to other of the many known stimuli of ET-1 secretion.

Interestingly, we observed increased *Ece1* mRNA expression in the hearts of mice fed a high-fat diet. This increase may have been partly mediated by the Ang II-NOX2 axis, as these mice had higher plasma levels of Ang II and increased cardiac expression of *Nox2* mRNA [11]. The involvement of this pathway could also be inferred from the fact that an increase in cardiac *Ece1* expression did not develop in *Nox2*-deficient mice. However, factors other than Ang II may also have contributed to the regulation of *Ece1* expression in response to a high-fat diet. These include native and oxidized low-density lipoproteins (nLDL and oxLDL, respectively). We previously found that both nLDL and oxLDL recruited Oct-1 and strongly activated the *NOX2* promoter in HMECs [11]. Another in vitro study demonstrated that nLDL and oxLDL increased dose-dependently *ECE1* mRNA in human endothelial vein endothelial cells (HUVECs) [12]. In vivo, an increased presence of ECE-1 was detected in human atherosclerotic plaques, although it seemed to predominate in smooth muscle cells and macrophages, rather than in endothelial cells [13]. In this respect, it has been suggested that while normally ECE-1 is expressed mainly by endothelial cells, it may also appear in other cell types after vessel injury and endothelial cell damage [13]. Also, given the large heterogeneity of endothelial cells [14,15], the impact of Ang II and other stimuli on endothelial cells may differ across various vascular beds. For example, while we observed an Ang II-induced increase in *ECE1* mRNA in endothelial cells from microvessels, such an effect of Ang II was not detected in HUVECs [16].

Our findings that the increase in *ECE1* expression in response to Ang II in vitro and high-diet in vivo can be reduced by either NOX2 inhibition or neutralization of superoxide anions further support earlier observations on the role of oxidative stress in vascular pathologies [17,18,19]. In contrast, the function of Oct-1 in regulating *ECE-1* gene transcription has not been reported before. Thus, our finding adds to the growing list of genes involved in development, homeostasis, and signal response that are controlled by Oct-1. Oct-1 executes these effects by binding the octamer DNA motif (5’-ATGCAAAT-3’) in gene promoters and by cooperating with other proteins to recruit and initiate transcription by RNA polymerase II [20]. In endothelial cells, genes known to be regulated by Oct-1 include *LOX1* that encodes a receptor for oxLDL [21,22] and *NOX4* that encodes superoxide anion-generating NADPH oxidase [23].

While NOX2-derived superoxide anions may act to promote *ECE1* transcription, it has also been demonstrated that superoxide can diminish ECE-1 activity by removing zinc from the enzyme [24]. Thus, one may hypothesize that it is a safeguard mechanism protecting against excessive EP-1 release. Although there is a rationale for inhibiting ECE-1 to ameliorate the adverse effects of Ang II in the cardiovascular system [25], ECE-1 inhibitors have not yet been translated into routine clinical applications. By deciphering how Ang II regulates *ECE1* expression in the endothelium, we add to our knowledge that may help minimize the untoward effects of ET-1 in disease.

## 5. Conclusions

In addition to directly inducing *ET-1* expression in endothelial cells, Ang II further promotes the release of mature ET-1 by stimulating *ECE1* expression and activity through a mechanism that involves the transcription factor Oct-1 and the superoxide anion-generating NOX2.

## Figures and Tables

**Table 1:** PCR primers used.

Gene	Gene-ID	Sense Primer 5′→3′	Anti-Sense Primer 5′→3′
Mouse *ECE-1*	NM_199307.1	gCAAAACAAgCTCCTTCCTg	TggCTgATCTCCgAgTCTCT
Mouse *GAPDH*	NM_008084.2	CATCACCATCTTCCAGGAGC	TGACCTTGCCCACAGCCTTG
Human *ECE-1*	NM_001397.2	CAAgCTCCTTCCTTgACCAg	gCCCAggTTgTTTTCTgTgT
Human *GAPDH*	AF261085.1	CATCACCATCTTCCAggAgCg	TgACCTTgCCCACAgCCTTg
Human *B2M*	NM_004048.2	GTGCTCGCGCTACTCTCTCT	CGGCAGGCATACTCATCTTT
pLuc 2000	NC_000001.11	TGGCCTAACTGGCCGGTACCACCTGGGCAAGGGTTGCAGTC	TCTTGATATCCTCGAGTGCCACCCGCGGCACCGCTGC
pLuc 1508	NC_000001.11	TGGCCTAACTGGCCGGTACCTACAACAGGGACACCACATTT	Identical sequence as pLuc 2000 above
pLuc 1008	NC_000001.11	TGGCCTAACTGGCCGGTACCACAGACACACGGCAACAAACC	Identical sequence as pLuc 2000 above
pLuc 608	NC_000001.11	TGGCCTAACTGGCCGGTACCCCTCCCACCGTTTCTGTCTCC	Identical sequence as pLuc 2000 above
pLuc 108	NC_000001.11	TGGCCTAACTGGCCGGTACCAGGCAGCCGAGCCGTCCGAGC	Identical sequence as pLuc 2000 above
EMSA	NC_000001.11	CAAATCCCAAATATAGTCAGGACT	AGTCCTGACTATATTTGGGATTTG

## Data Availability

Data available on request.

## Supplementary Materials

The following supporting information can be downloaded at: https://www.mdpi.com/article/10.3390/antiox13040500/s1. Supplementary Figure S1. The effect of NOX2-siRNA on NOX2 and GAPDH protein expression. Supplementary Figure S2. The effect of NOX1- and NOX4-targeting siRNAs on the expression of respective mRNAs and proteins.
