# Supplementary material for: The Role of NOX2-Derived Reactive Oxygen Species in the Induction of Endothelin-Converting Enzyme-1 by Angiotensin II
Source: Antioxidants (Basel). 2024 Apr 22;13(4):500. doi: 10.3390/antiox13040500 (PMC11047448; doi:10.3390/antiox13040500)

Suppl Fig. S1.

The effect of *NOX2*-siRNA on NOX2 and GAPDH protein expression.
HMECs were transiently transfected with *NOX2*-siRNA at doses indicated and then stimulated with Ang II (1 μmol/L) for 12 hours. The expression of NOX2 and GAPDH proteins was analyzed by Western blotting. Results of 2 independent experiments are presented on original uncropped gels and as quantified normalized data

The order of samples is as follows:

(M) molecular weight marker;
(1 and 7) Ang II;
(2 and 8) Ang II + *NOX2*-siRNA (10 nM),
(3 and 9) Ang II + *NOX2*-siRNA (1 nM),
(4 and 10) Ang II + *NOX2*-siRNA (0.1 nM),
(5 and 11) Ang II + *NOX2*-siRNA (0.01 nM),
(6) Ang II + *NOX2*-siRNA (0.001 nM).

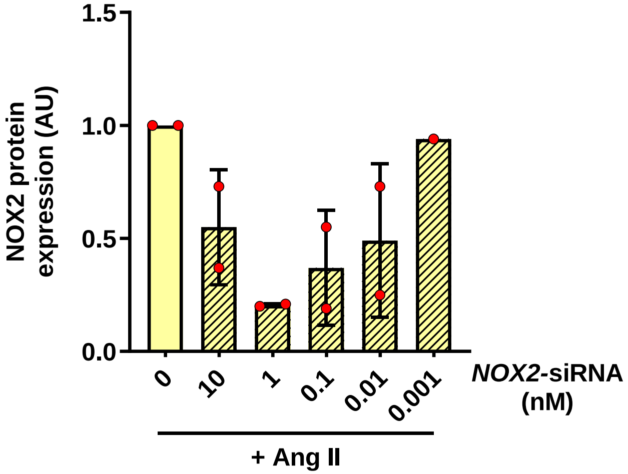

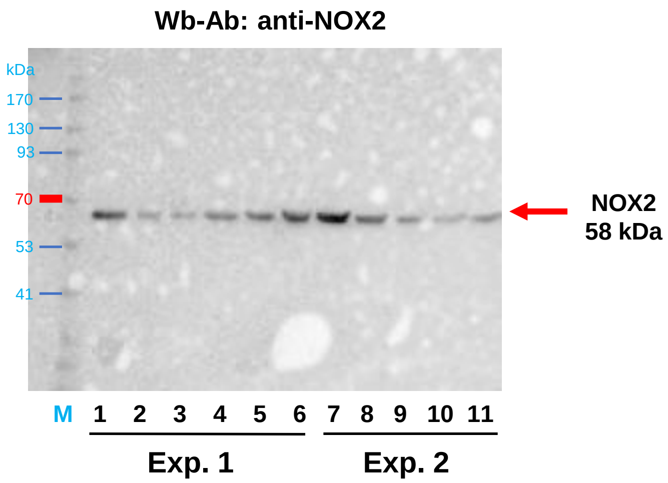

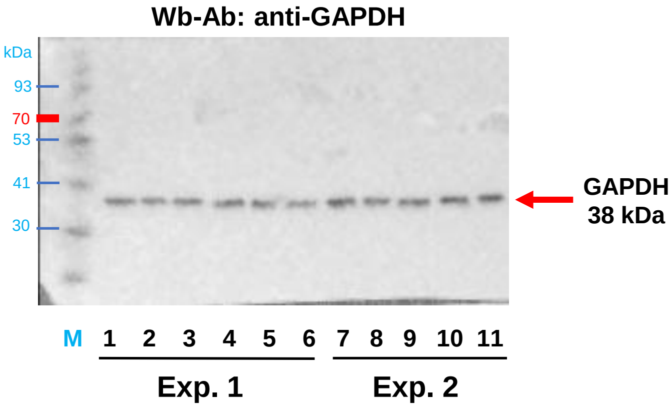

Suppl Fig. S2.

The effect of *NOX1*- and *NOX4*-targeting siRNAs on the expression of respective mRNAs and proteins.
HMECs were transiently transfected with *NOX1*- and *NOX4*-siRNAs at doses as indicated and then stimulated with Ang II (1 µmol/L) for 12 hours. The expression of target mRNAs and proteins was analyzed by RT-qPCR (n=4) and Western blotting (n=2), respectively. Original uncropped gels from Western blots are presented. The order of samples was as follows: (M) molecular weight marker; (1 and 6) control; (2 and 7) Ang II; (3 and 8) Ang II + *NOX*-siRNA (0.1 nM), (4 and 9) Ang II + *NOX*-siRNA (1 nM), (5 and 10) Ang II + *NOX*-siRNA (10 nM).

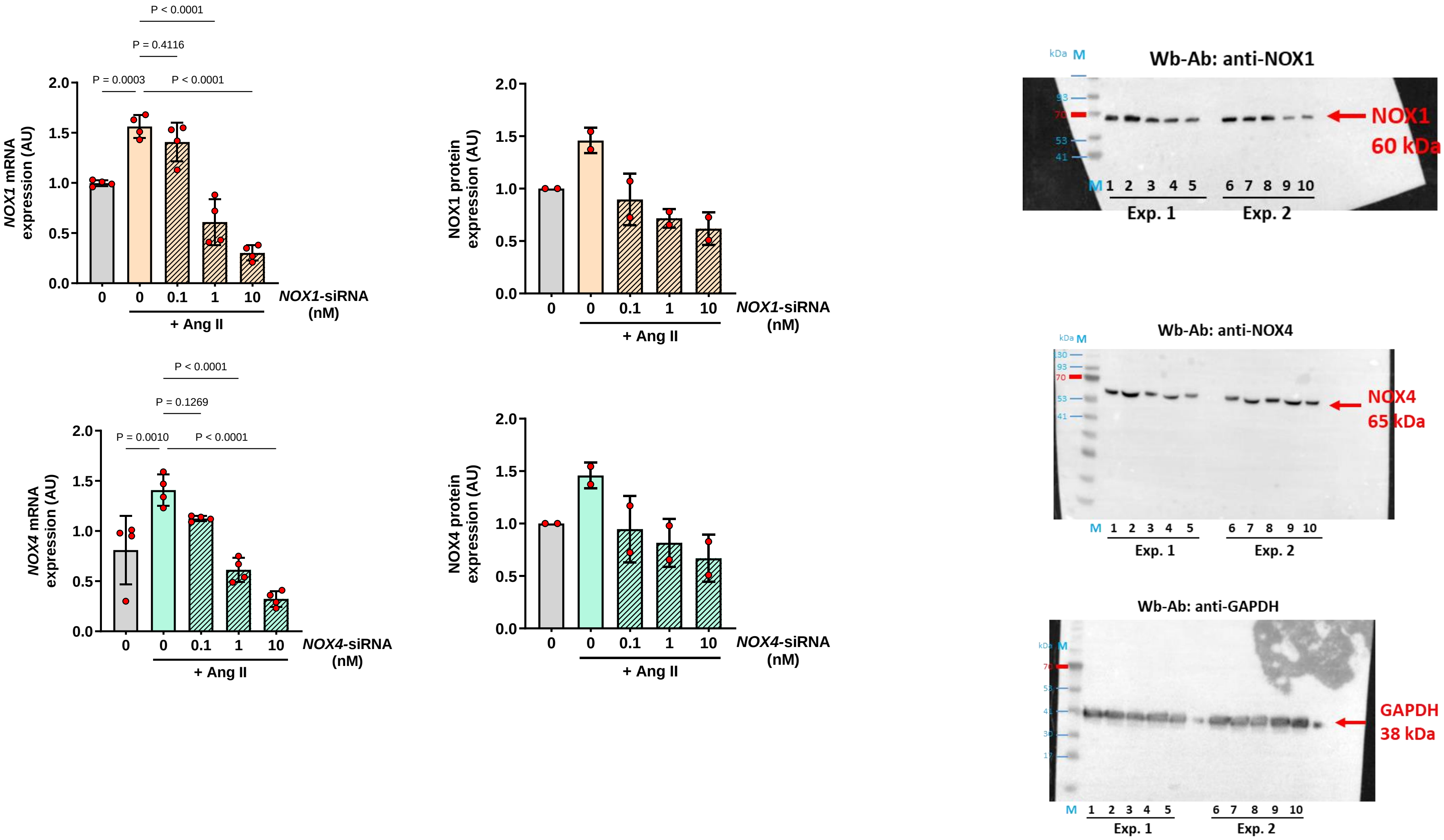

Supplement: Supplementary file 1 [file antioxidants-13-00500-s001.zip › antioxidants-2962702-supplementary.pdf]
